# Phenotypic and Genomic Characteristics of *Campylobacter gastrosuis* sp. nov. Isolated from the Stomachs of Pigs in Beijing

**DOI:** 10.3390/microorganisms11092278

**Published:** 2023-09-10

**Authors:** Hairui Wang, Yixin Gu, Lihua He, Lu Sun, Guilan Zhou, Xiaoli Chen, Xin Zhang, Zhujun Shao, Jianzhong Zhang, Maojun Zhang

**Affiliations:** State Key Laboratory for Infectious Disease Prevention and Control, National Institute for Communicable Disease Control and Prevention, Chinese Center for Disease Control and Prevention, Beijing 102206, China

**Keywords:** *Campylobacter gastrosuis*, novel species, genomic characteristics, phylogenetic analyses, antibiotic resistance

## Abstract

*Campylobacter* is among the four main causes of gastroenteritis worldwide. Most reported *Campylobacter* infections are caused by *C. jejuni* and *C. coli*. However, other emerging *Campylobacter* pathogens have been recognized as important pathogens in humans and animals. A novel bacterial strain, PS10^T^, was isolated from the gastric mucous of pigs in 2022 in Beijing, China. The cell was Gram-negative, microaerobic, motile, and negative for catalase, oxidase, and urease. Phylogenetic and phylogenomic analyses based on the 16S rRNA gene and core genome indicated that this isolate belongs to the genus *Campylobacter*. There were low dDDH relatedness and ANI values shared within this strain and its closest species *C. mucosalis* below the cut-off values generally recognized for isolates of the same species. The draft genome size of PS10^T^ is 2,240,910 bp in length with a percentage of DNA G+C contents of 37.72%. Comparing the phenotypic and phylogenetic features among this isolate and its related organisms, strain PS10^T^ represents a novel species within the genus *Campylobacter*, for which the name *Campylobacter gastrosuis* sp. nov. (Type strain PS10^T^ = GDMCC 1.3686^T^ = JCM 35849^T^) is proposed.

## 1. Introduction

The genus *Campylobacter* belongs to the family Campylobacteraceae and the order Campylobacterales, and they currently have over 50 species and subspecies, including many non-validly described species, which have been isolated from different times and sources according to the List of Prokaryotic names with Standing in Nomenclature (LPSN, https://lpsn.dsmz.de/genus/campylobacter) (accessed on 20 June 2023). Members of the *Campylobacter* genus are nutritionally fastidious and strictly grow under anaerobic or microaerobic conditions, and they are morphologically diverse, including spiral-, curved-, or rod-shaped. *Campylobacter* naturally colonizes humans, other mammals, birds, reptiles, and shellfish, particularly birds, and can be transmitted to humans through contaminated food or water [1,2,3,4].

*Campylobacter* is among the four main causes of gastroenteritis worldwide [5]. Most reported *Campylobacter* infections are caused by *C. jejuni*, which is a leading cause of bacterial gastroenteritis in humans, and symptoms typically include diarrhea, abdominal cramping, and fever, amongst others [6,7]. In addition, the antecedent infection of *C. jejuni* could trigger a Guillain–Barré Syndrome (GBS) outbreak [8]. And, to a lesser extent, *C. coli* accounts for 1–25% of all *Campylobacter*-related diarrheal diseases [9]. However, other emerging *Campylobacter* pathogens have been recognized as important pathogens in humans and animals [10]. *C. upsaliensis* has been isolated from patients with diarrhea, bacteremia, hemolytic uremic syndrome, and those who have undergone an abortion [5,11]. *C. lari* causes not only sporadic gastrointestinal infections in humans but also water outbreaks and bacteremia, and occasionally suppurative pleurisy, reactive arthritis, prosthesis, and urinary tract infections [5,12,13,14,15]. Therefore, the emerging *Campylobacter* pathogens and their pathogenicity to humans or animals need to be further discovered and studied.

Antimicrobial resistance in *Campylobacter* is a pressing concern, complicating the clinical treatment of infections caused by this bacterium. The prevalence of resistance to commonly used antibiotics of choice for the treatment of human campylobacteriosis, like fluoroquinolones, macrolides, and tetracyclines, is high, and multidrug-resistant strains are on the rise, meaning there is a risk of resistant strains spreading from animals to human, posing a significant threat to public health [16,17,18,19]. Notably, new antibiotic resistance mechanisms are continuously emerging in *Campylobacter* [20]. As such, continued monitoring and surveillance of *Campylobacter* antimicrobial resistance patterns is crucial to inform effective treatment strategies and to curb the rise and spread of resistance [20,21].

Poultry is the main source of human campylobacteriosis, and the pig is also considered an important source of *C. coli*-related human campylobacteriosis [22]. The study has found a possible link between human campylobacteriosis and pig-borne *Campylobacter* [23]. Moreover, *Campylobacter* spp. from pigs was found, possibly propagated in each slaughtering step, to have an average prevalence of 19.3% [24] and a high prevalence of resistance to multiple antibiotics, particularly macrolides, which is probably attributable to the overuse of antimicrobials in pig production [25]. As pigs are usually subclinically infected, the carcasses could have a high chance of being contaminated by *Campylobacter* spp. during the slaughter process, which can be transmitted to humans via contaminated pork [26].

This study described the phenotypic, taxonomic, antimicrobial susceptibility, and genomic characteristics of a novel *Campylobacter*-like isolate from the gastric mucous of pigs, and the phylogenetic and phylogenomic relationships between the isolated strain and other *Campylobacter* species were also clarified. Based on polyphasic taxonomic analyses, this novel isolate is proposed as a novel *Campylobacter* species, designated *Campylobacter gastrosuis* sp. nov. (PS10^T^).

## 2. Methods

### 2.1. Sampling, Isolation, and Culturing

In the exploration of *Campylobacter* spp. diversity in animals, the isolation process was executed utilizing the *Campylobacter* isolation kit (ZC-CAMPY-002, Qingdao Sinova Biotechnology Co., Ltd., Qingdao, China), which incorporates a membrane filter method. In a succinct overview, 0.4 mL of pig gastric mucous was added to 4 mL of enrichment buffer from the kit. The enriched suspension was then incubated at 37 °C for 24 h within a microaerophilic environment comprising 5% O_2_, 10% CO_2_, and 85% N_2_. Following this, approximately 300 μL of the cultured enrichment suspension was applied onto the filter’s surface affixed to the double medium plates. These plates contained Karmali and Columbia agar, each with 5% defibrinated sheep blood. Subsequently, the medium plates were placed within a microaerophilic atmosphere at 37 °C and incubated for 48 h [27].

At least 5 individual colonies exhibiting potential *Campylobacter* genus traits were carefully selected for Gram staining, subsequent passage cultivation, and purification. Those isolates that exhibited morphology consistent with *Campylobacter* were then subjected to initial characterization by PCR amplification and sequencing of the 16S rRNA gene [28]. Following this, the selected isolates were preserved at −80 °C in BHI medium containing 20% (*v*/*v*) glycerol, facilitating further analyses.

### 2.2. Morphological, Physiological, and Biochemical Characteristics

For the investigation of morphological and biochemical attributes, cells were cultivated and harvested during the late exponential growth phase. Gram staining was performed using a Gram staining kit (Baso) [29], and the samples were observed under a light microscope (Eclipse Ci-L, NIKON). Biochemical tests were conducted to elucidate the physiology and chemotaxonomy of this isolate. The catalase activity was assessed using a 3% (*v*/*v*) H_2_O_2_ solution to observe bubble production. Further biochemical characteristics specific to *Campylobacter* spp. were determined using the API Campy identification system (bio-Mérieux), adhering strictly to the manufacturer’s instructions. As a benchmark, *C. jejuni* ATCC 33560^T^ was utilized as a control for comparison.

### 2.3. Antimicrobial Susceptibility Testing

The minimum inhibitory concentrations (MICs) for eleven antimicrobials (erythromycin, azithromycin, nalidixic acid, ciprofloxacin, gentamicin, streptomycin, chloramphenicol, florfenicol, tetracycline, telithromycin, and clindamycin) were determined for this *Campylobacter* isolate using the gradient strip diffusion method (E-test, bio-Mérieux) in accordance with the manufacturer’s instructions, as previously reported [30]. After 48 h of growth on Karmali blood agar plates, the isolate was suspended in sterile saline and adjusted to a McFarland turbidity standard of 1. The bacterial suspension was then evenly spread onto Karmali blood agar plates using a sterile cotton swab. Plates containing E-test strips were subsequently incubated under microaerobic conditions for 48 h. The MIC was determined as the lowest concentration that did not exhibit visible growth. The type strain *C. jejuni* ATCC 33560^T^ was employed as the control.

### 2.4. Genome Extraction and Sequencing

Subsequent to cultivation, genomic DNA was extracted employing the QIAamp DNA Mini Kit (Qiagen, Hilden, Germany), adhering to the manufacturer’s guidelines. The concentration and purity of the DNA samples were assessed using the NanoDrop spectrophotometer (Thermo Scientific, Waltham, MA, USA). Quality criteria encompassed a concentration of ≥20 ng/μL and a total amount exceeding 2 μg. Additionally, a purity requirement dictated that the OD260/OD280 value should fall within the range of 1.6 to 1.8.

DNA sequencing was conducted using the Illumina PE150 platform (Illumina Inc., San Diego, CA, USA) at the Novogene Corporation (Beijing, China), with coverage reaching a depth of 100×. A 350 bp paired-end library was established to facilitate genome sequencing, yielding 150 bp reads. To appraise and enhance the quality of raw sequence data, FastQC 0.11.9 [31] and fastp v0.23.4 [32] software tools were employed. Low-quality reads, defined by a sequence quality score ≤ Q30 across ≥3 consecutive bases, were subsequently excluded. The resultant high-quality reads were then assembled using SOAPdenovo2 [33].

### 2.5. Phylogenetic and Phylogenomic Analysis

To ascertain the strain’s phylogenetic placement, we initiated PCR amplification of the 16S rRNA gene. Following amplification, each PCR product underwent purification and subsequent subcloning into the pMD18-T vector. Subsequently, the vector was introduced into *Escherichia coli* DH5α, and the inserted 16S rRNA gene fragment was extracted from a single colony post-lysis and subjected to sequencing.

The resulting 16S rRNA gene sequences were then cross-referenced with other *Campylobacter* species using EzBioCloud’s identification service, enabling the taxonomic classification of this strain [34]. Employing the MAFFT 7.471 software [35], a multiple sequence alignment was performed on the 16S rRNA gene sequences of this strain and the type strains within the *Campylobacter* genus. Phylogenetic analysis ensued utilizing the MEGA X [36] software package, employing the neighbor-joining (NJ) [37], maximum-parsimony (MP) [38], and maximum-likelihood (ML) algorithms [39], all supplemented with a bootstrap analysis comprising 1000 replicates [40]. Furthermore, the type strain of *Arcobacter butzleri* ATCC 49616^T^ was incorporated as an outgroup reference.

The assembled sequences underwent gene prediction and functional annotation using Prokka 1.14.6 software [41] and the tRNAscan-SE 2.0 tool [42]. The protein sequences of core genes sourced from this isolate and other *Campylobacter* species were extracted, leveraging CD-HIT 4.8.1 software [43] and .faa files from the Prokka results. The extraction was based on a 40% protein sequence similarity threshold, followed by alignment using MAFFT 7.471 software. Subsequently, the phylogenomic tree was constructed via FastTree 2.1.10 [44], and its visualization was facilitated using Dendroscope 3.8.3 software [45].

### 2.6. Genomic Analysis

The sequences of predicted proteins underwent assignment and annotation to the Clusters of Orthologous Groups (COGs) database through eggNOG-mapper v2 [46]. Genomic island and plasmid predictions for the isolate were performed using the online tools IslandViewer 4 [47] and PlasmidFinder 2.0 server [48], respectively. To identify prophage sequences, both the Phage Search Tool Enhanced Release (PHASTER) web server [49] and phiSpy 4.2.21 software [50] were employed. The Comprehensive Antibiotic Resistance Database (CARD) 3.2.7 and its Resistance Gene Identifier (RGI) 6.0.2 [51] were utilized for screening antimicrobial resistance genes and related mutations. Comparative analysis of antibiotic resistance gene clusters was carried out using Easfig v2.2.5 [52]. The VFanalyzer [53] was used to detect virulence genes in all genomes. Digital DNA-DNA hybridization (dDDH) relatedness calculations and comparisons were performed using the Genome-to-Genome Distance Calculator 3.0 [54]. Additionally, average nucleotide identity (ANI) values were determined using Pyani 0.2.10 software [55]. The visualization of COG classification, genomic islands, and virulence genes results was achieved using the ggplot2, genoPlotR, and pheatmap packages within R 4.2.2, respectively.

## 3. Results and Discussion

### 3.1. Isolation and Phenotypic Characterization

In 2022, a total of 20 *Campylobacter* isolates, like *C. coli*, *C. fetus*, *C. hyointestinalis,* and others, were isolated from 19 health gastric mucous samples collected from a pig slaughterhouse from Beijing, China (20/19, 105.26%). The strain PS10^T^ is one of these isolates. This PS10^T^ cell is Gram-negative, microaerobic, motile, and spiral-shaped (Figure S1). The colonies were circular, 2–3 mm in diameter, smooth, and grey after 2 days of growth on Karmali agar with 5% defibrinated sheep blood. Cells appear coccoid after 5–6 days of incubation or when exposed to air. No hemolysis was observed on the blood agar. The colony and morphological outcomes of this strain remained consistent with the fundamental attributes associated with *Campylobacter* [56].

The phenotypic and biochemical characteristics of PS10^T^ exhibited distinct traits that set it apart from the standard profile of any other species within the *Campylobacter* genus. Notably, this strain tested negative for catalase, oxidase, and urease activities. Of particular significance is that the absence of oxidase activity is a trait shared with only a limited number of other members within the *Campylobacter* genus, such as *C. gracilis* and *C. ornithocola* [57], which makes it recognizable from closely related species *C. mucosalis*. The absence of H_2_S production further contributes to its distinctiveness from closely related species *C. mucosalis*. The type strain of this novel species also stands out due to its inability to hydrolyze hippurate, while it exhibits the capability to hydrolyze indoxyl acetate and reduce nitrate, setting it apart from *C. suis*. Finally, PS10^T^ could be unequivocally differentiated from *C. mucosalis* due to its lack of oxidase activity and inability to generate H_2_S (Table 1) [57,58,59,60]. In the realm of biochemical phenotype, isolate PS10^T^ showcases distinctive attributes within the *Campylobacter* genus, allowing for its precise differentiation from other *Campylobacter* species through its unique biochemical profiles.

### 3.2. Phylogenetic and Phylogenomic Analysis

The assessment against the EzTaxon-e database of nearly full-length 16S rRNA gene sequences (1510 bp) indicated that this isolate exhibited its closest affiliation with the representative specimen of the *Campylobacter* genus (Domain, Bacteria; Phylum, Pseudomonadota; Class, Epsilonproteobacteria; Order, Campylobacterales; Family, Campylobacteraceae). Strain PS10^T^ was closest to *C. mucosalis* DSM 21682^T^ (98.81% of 16S rRNA gene identity). The value of similarity was slightly higher than 98.70%,which was the generally accepted threshold for the species [61]. Thus, if no further identification methods were employed beyond the conventional 16S rRNA sequence comparison, this isolate could potentially be classified as *C. mucosalis* based on existing experience.

**Figure 1:**
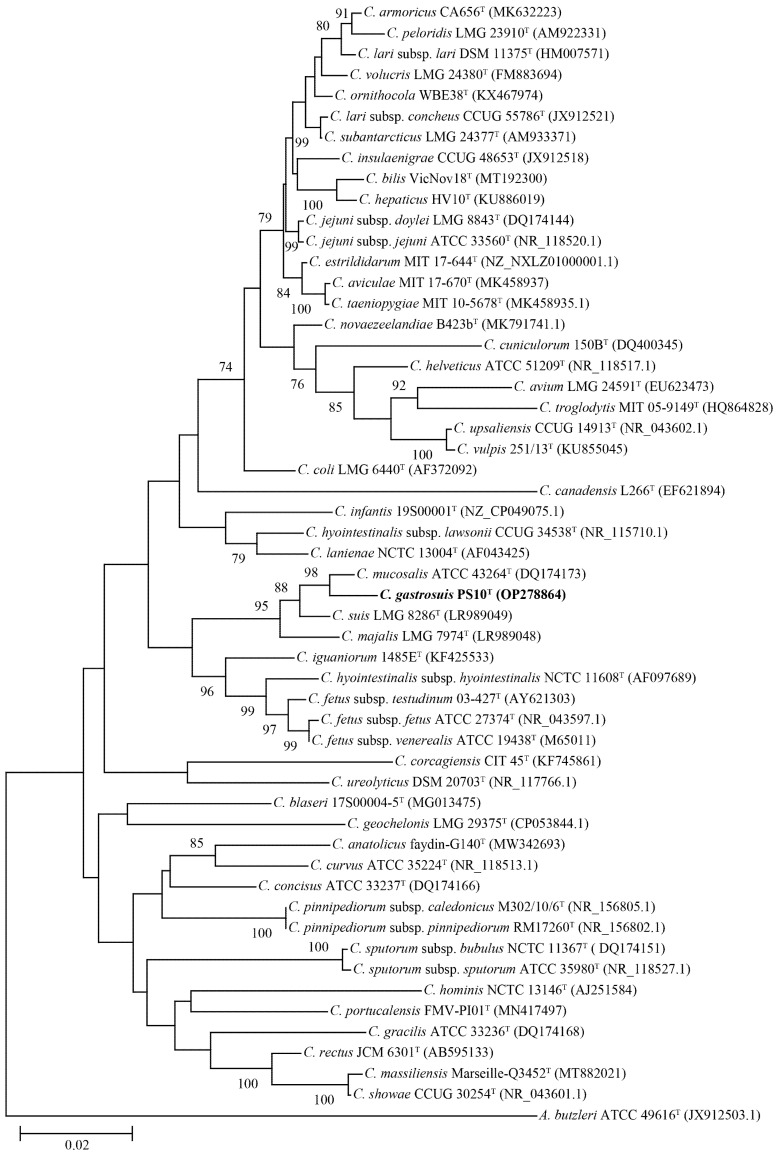
Neighbor-joining phylogenetic tree based on nearly complete 16S rRNA gene showing the relationships between our isolates and the type strains of the genus *Campylobacter*. Bootstrap values (>70%) based on 1000 replicates are shown at branch nodes, with *Arcobacter butzleri* ATCC 49616^T^ as an outgroup. Bar, 0.02 changes per nucleotide position. Novel strain is highlighted in bold.

The phylogenetic tree constructed using the NJ algorithm (as shown in Figure 1) and based on the nearly complete 16S rRNA gene sequences demonstrated that PS10^T^ is positioned within the *Campylobacter* genus. Notably, this strain, alongside *C. mucosalis* ATCC 43264^T^, *C. majalis* LMG 7974^T^, and *C. suis* LMG 8286^T^, formed a distinct cluster that exhibited a certain degree of independence. Among these strains, *C. mucosalis* ATCC 43264^T^ emerged as the closest relative. Moreover, the phylogenetic trees generated using the ML and MP algorithms produced comparable topological outcomes, further corroborating these findings (Supplementary data Figures S2 and S3).

**Figure 2:**
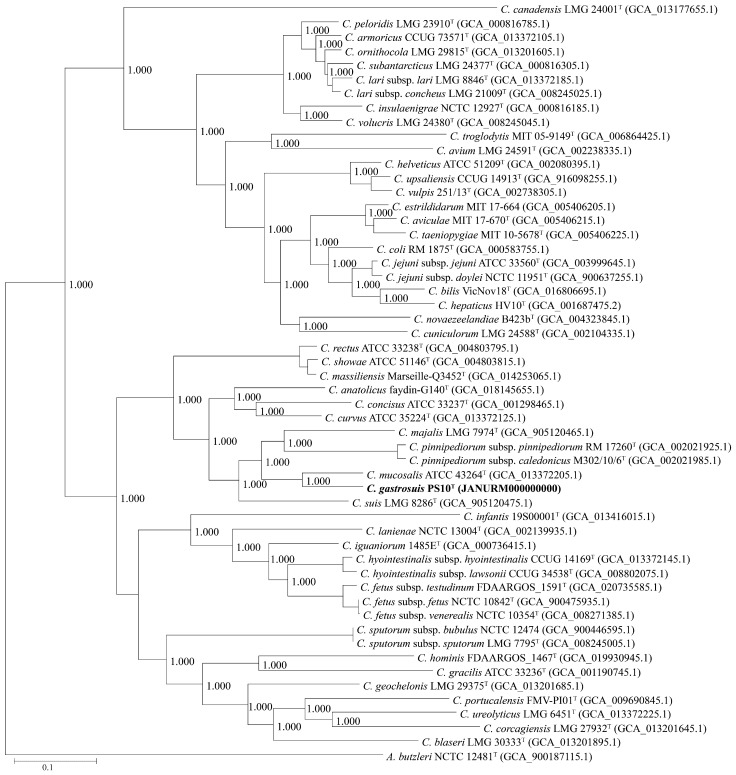
Neighbor-joining phylogenomic tree based on 332 core genes of the genus *Campylobacter*. The outgroup is *Arcobacter butzleri* ATCC 49616^T^. Novel strain is highlighted in bold.

Utilizing a protein identity threshold of 40%, a set of 344 core genes, considered orthologous groups, were extracted and shared across this isolate and all accessible genomes of other species within the *Campylobacter* genus. This collection of core genes was then employed to construct a phylogenomic tree at the genome level (depicted in Figure 2). Analogous to the phylogenetic tree rooted in 16S rRNA gene sequences, PS10^T^ was again positioned within an independent cluster alongside *C. mucosalis* ATCC 43264^T^, *C. majalis* LMG 7974^T^, and *C. suis* LMG 8286^T^. Notably, this cluster also encompassed strains *C. pinnipediorum* subsp. *pinnipediorum* RM 17260^T^ and *C. pinnipediorum* subsp. *caledonicus* M302/10/6^T^. These findings collectively reinforce the classification of this isolate within the *Campylobacter* genus. Importantly, the consistent outcome underscores *C. mucosalis* as its closest species.

The amalgamation of outcomes from the comparison of 16S rRNA gene sequences, alongside the analyses performed at both phylogenetic and phylogenomic levels, unequivocally categorizes this isolate within the *Campylobacter* genus. However, due to its placement within a cluster housing several other *Campylobacter* species, notably *C. mucosalis*, the strain’s identification at the species level remains somewhat challenging. As a result, additional methods are warranted to discern this strain’s specific species designation with greater accuracy.

### 3.3. Genome Characteristics

The final genome assembly of strain PS10^T^ contained 59 contigs with a draft genome size of 2,240,910 bp and a genomic GC content of 37.72%, which is slightly higher than the most closely related bacterium, *C. mucosalis* ATCC 43264^T^ (GC content, 36.55%), and within the range of DNA base compositions previously reported for the members in the genus *Campylobacter* (GC content, 29–47%) [62]. The Prokka predicted 2325 coding genes in total with the draft genome, among which 1186 (51.01%) belong to predicted proteins and 1139 (48.99%) were assigned as hypothetical proteins. The genome contained 43 tRNAs, a tmRNA, a 16S rRNA, and a 23S rRNA. A total of three insertion sequence (IS) elements were found, namely IS*1595*, IS*1380*, and IS*4*. However, the presence of plasmids, phages, and phage-like elements was not predicted in the draft genome.

**Figure 3:**
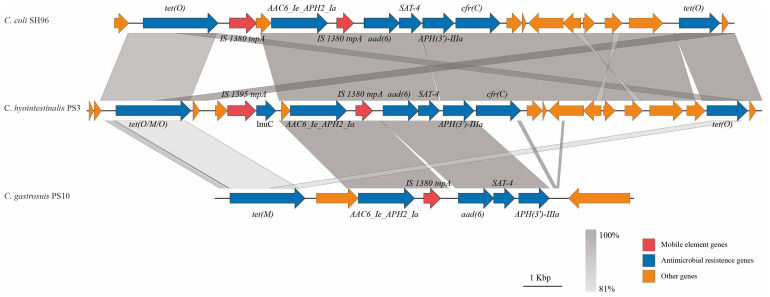
Genetic environments of MDRGIs in *Campylobacter* isolates. Arrows indicate the direction of transcription of the genes, and different genes are shown in different colors. IS, insertion sequence.

Moreover, a multi-drug resistance genomic island (MDRGI) was predicted, and it monopolizes a scaffold on the draft genome of this strain. The MDRGI contains a *tet(M)* gene, an *AAC6_Ie_APH2_Ia* gene, and an *aad(6)-SAT-4-APH(3′)-IIIa* gene cluster (Figure 3). These antibiotic resistance correlative genes could confer resistance to the antibiotics of tetracycline, nucleoside, and aminoglycoside. This MDRGI was in an independent genome contig, consisting of 10,736 bp bases, and the GC content was 33.7%, which was significantly lower than that of the draft genome of PS10^T^ (37.72%), indicating that this fragment may be exogenously obtained from the other species [63]. Meanwhile, a predicted insertion sequence, IS*1380*, was distributed across this MDRGI. This type of MDRGI was shorter than that from PS3 (17,302 bp), another strain of *C. hyointestinalis* isolated in this project, and SH96 (15,885 bp), a strain of *C. coli* isolated from fecal samples of pigs [64]. The antibiotic resistance correlative genes in PS10^T^ were also present in the other two strains, and both of them were *AAC6_Ie_APH2_Ia* gene and *aad(6)-SAT-4-APH(3′)-IIIa* gene cluster distributed at the ends of IS*1380*. In 2015, the isolation rate of *aad(6)-SAT-4-APH(3′)-IIIa* gene cluster in streptomycin-resistant strains has reached 10.86% [65]. However, the MDRGI in the SH96 strain was located on the plasmid, indicating that it may be horizontally transferred on chromosomes or plasmids between different strains as a whole and undergo evolutionary mutations and lead to receptor strains’ resistance to tetracycline, nucleoside, and aminoglycoside. The horizontal gene transfer (HGT) between host bacterial cells relies on the transfer of genetic material via mobile genetic elements (MGEs), such as phages, plasmids, transposons, and integrons [66,67]. These critical genes may facilitate the drug-resistant MGEs, particularly plasmids, which can be transmitted between bacterial populations [68]. Due to the lack of a strict restriction enzyme modification system, *Campylobacter* has a high mutation rate and easy access to exogenous genetic elements, which could lead to the acquisition or loss of antibiotic resistance correlative elements [69].

**Figure 4:**
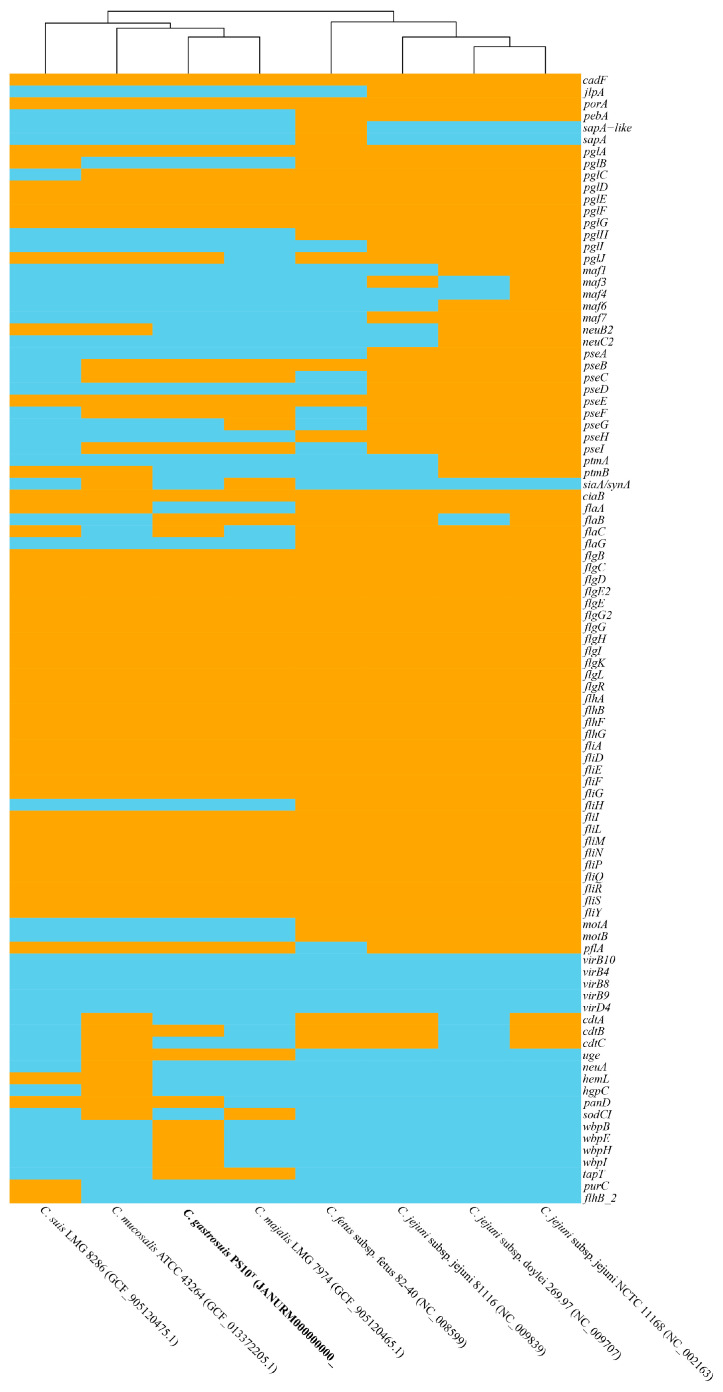
Heatmap of the distribution of virulence genes. Orange indicates the presence of virulence genes, and sky blue indicates the absence of virulence genes. Novel strain is highlighted in bold.

In genomes of PS10^T^, numerous *Campylobacter* virulence-associated genes were detected, which could encode genes related to adherence, colonization and immune evasion, glycosylation, invasion, motility and export apparatus (flagella and chemotaxis), secretion system, toxin, and other virulence factors. The virulence-associated gene profile of this strain is similar to that of *C. mucosalis* ATCC 43264^T^, *C. majalis* LMG 7974^T^, and *C. suis* LMG 8286^T^, mainly related to glycosylation, motility, and export apparatus. This type strain of PS10^T^ has the incomplete cytolethal distending toxin (CDT, genes coding for the three subunits: *cdtA*, *cdtB*, and *cdtC*), while *C. mucosalis* ATCC 43264^T^ has the complete CDT (Figure 4). And both PS10^T^ with the other closely relative strain did not hold the type IV secretion system (T4SS) and iron uptake system (*fur* and *cfrA* genes). However, further experimental studies should confirm whether PS10^T^ is a pathogen to the host. In silico screening results of the presence or absence of these virulence genes are presented in Figure 4.

**Figure 5:**
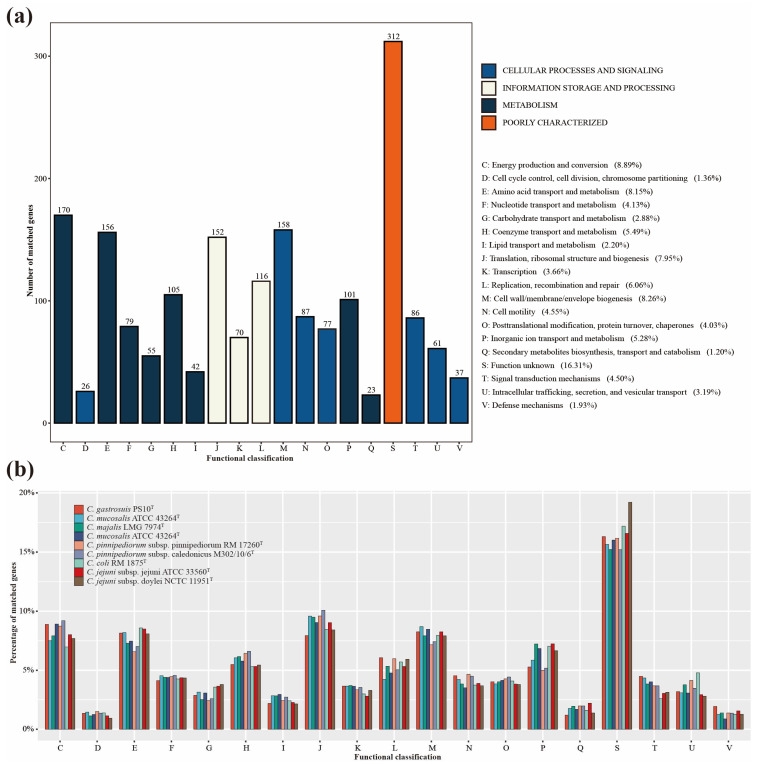
COG functional classification. (**a**) COG functional classification of genes belonging to PS10^T^. (**b**) COG functional classification of genes belonging to PS10^T^ and closely related *Campylobacter* species.

By employing the COG database, a total of 1601 proteins were successfully assigned functional classifications, while 312 remained unclassified (as depicted in Figure 5a). Specifically, the number of proteins attributed to “cellular processes and signaling”, “information storage and processing”, and “metabolism” was 532, 338, and 731, respectively. In comparison with closely related species on the phylogenomic tree and significant pathogenic species within the *Campylobacter* genus, PS10^T^ exhibited a slight abundance of proteins within “Defense mechanisms” and “Signal transduction mechanisms”, both categorized under “cellular processes and signaling”. Conversely, it showcased a minor reduction in proteins for categories like “Lipid transport and metabolism”, “Translation, ribosomal structure, and biogenesis”, “Inorganic ion transport and metabolism”, and “Secondary metabolites biosynthesis, transport, and catabolism” (as depicted in Figure 5b). In alignment with the outcomes of the phylogenetic and phylogenomic analysis, the findings from the analysis of the COG database demonstrated a congruence in the annotation results between PS10^T^ and its closely related species, exhibiting no notable differences.

The dDDH scores of this isolate with the other species in the genus *Campylobacter* were below 70%, the threshold for species demarcation [54]. Meanwhile, as the gold standard for the delineation of bacterial species, the ANI values between this isolate and all established species of *Campylobacter* were below 95%, the cutoff for species demarcation (Table 2 and Table S1) [70]. These results suggested that strain PS10^T^ represented a novel species of the genus *Campylobacter*. Although 16S rRNA sequencing is widely used for strain identification, it could not accurately identify the correct species of PS10^T^, and the identification at the whole genome level is accurate. With the advancement of sequencing technology, microbial whole genome sequencing (WGS) technology has become the most effective means of bacterial species identification, and the gold standard of bacterial species identification has been updated from the DDH to the ANI [70]. In the current identifications of isolates, these genomic-level identification methods make up for the errors caused by the traditional identification of the 16S rRNA short sequence.

### 3.4. Antibiotic Resistance

Antibiotic resistance was demonstrated through the fact that strain PS10^T^ has high MIC values to five types of antibiotics, namely macrolides (erythromycin and azithromycin), quinolones (nalidixic acid), tetracyclines (tetracycline), ketolides (telithromycin), and lincosamides (clindamycin) (Table 3). A part of our results was consistent with previous reports showing that *Campylobacter* species are highly resistant to macrolides, quinolones, and tetracyclines [18,20,21,27,71]. Macrolides and fluoroquinolones have been the mainstays of therapy and are used in the production of agricultural animals. However, resistance into these antibiotics, particularly fluoroquinolones such as ciprofloxacin, is common [17,72]. *C. coli* was the predominant strain of *Campylobacter* isolated from the feces of pigs, and resistance to tetracycline was also common [25,57,73]. As foodborne pathogens, *Campylobacter* require continued monitoring and surveillance in antibiotic usage and the prevalence of antimicrobial resistance patterns.

In the PS10^T^ genome, in addition to the antibiotic resistance relative genes on the MDRGI, another *adeF* gene that was detected to mediate tetracycline resistance exists in another contig. However, there are large differences between resistance phenotypes and antibiotic resistance relative genes, and only the resistance of tetracycline is consistent among them. Although there were tetracycline, nucleoside, and aminoglycoside resistance genes on the MDRGI, the isolate did not exhibit corresponding phenotypes, which may be caused by the functional dormancy of this isolate [64]. On the other hand, tetracycline resistance may be mediated by the *adeF* gene, which is not present in the MDRGI. However, antibiotic resistance genes related to macrolides, quinolones, ketolides, and lincosamides were also not found in the genome, suggesting that further research about drug resistance mechanisms is needed to understand the resistance mechanism of these drugs.

The expression and activity of antibiotic resistance genes may be related to the status of bacteria, and drug-resistant MGEs may play an important role in the development of bacterial resistance as a risk factor for drug resistance. Therefore, it is necessary to detect the drug-resistant phenotype, drug-resistant related genes, and drug-resistant related gene mutations of pathogenic bacteria to monitor the resistance of pathogens.

## 4. Conclusions

Using a polyphasic approach, including DNA sequencing and analysis (16S rRNA gene and whole genome sequencing), and a wide range of phenotypic tests, as suggested by On et al. [56], provided sufficient evidence to distinguish this isolate from its closely related type strains and to confirm that this isolate represents a novel species in the genus *Campylobacter*. With PS10^T^ as the type strain, we suggest the name *Campylobacter gastrosuis* sp. nov. for this novel member of the genus *Campylobacter*. Meanwhile, we also tested the antibiotic sensitivity of this isolated strain and found that it was resistant to a variety of antibiotics commonly used in *Campylobacter*, and we found an MDRGI on the genome. Additional investigation into the pathogenesis, prevalence among pigs, as well as the potential for human infection through contact with pigs, is imperative. Such research could aid in better understanding and managing diseases attributed to this novel species. A description of *Campylobacter gastrosuis* sp. nov. is presented in Table 4.

## Figures and Tables

**Table 1:** Phenotypic characteristics of *Campylobacter gastrosuis* sp. nov. strain and the closely related *Campylobacter* species.

Characteristic	1	2	3	4	5	6
Catalase	−	−	−	−	+	+
Oxidase	−	+	−	−	−	+
Urease	−	−	−	−	+	+
Nitrate reduction	+	(−)	−	−	+	+
Indoxyl acetate hydrolysis	+	−	−	−	−	−
Hippurate hydrolysis	−	−	−	−	−	−
H_2_S	−	+	+	−	+	+

1, *C. gastrosuis* sp. nov.; 2, *C. mucosalis*; 3, *C.majalis*; 4, *C. suis*; 5, *C. pinnipediorum* subsp. *caledonicus*; 6, *C. pinnipediorum* subsp. *pinnipediorum*. Data for other species were taken from previous publications [57]. +, 90–100% positive; (−), 11–25% positive; −, 0–10% positive.

**Table 2:** ANI (lower diagonal) and dDDH (upper diagonal) among novel *Campylobacter* strains and closely related *Campylobacter* species.

	1	2	3	4	5	6	7	8	9
1	1	27.90%	19.10%	19.40%	20.20%	20.00%	20.00%	20.90%	19.00%
2	78.58%	1	71.37%	18.00%	19.50%	19.70%	66.17%	66.26%	66.35%
3	72.14%	20.80%	1	20.40%	18.40%	17.90%	66.60%	66.53%	66.71%
4	71.17%	72.82%	71.41%	1	70.36%	70.29%	65.96%	65.82%	65.90%
5	71.16%	71.02%	71.68%	17.90%	1	56.90%	67.60%	67.60%	67.61%
6	70.92%	71.04%	71.51%	19.30%	94.27%	1	67.78%	67.74%	67.92%
7	66.21%	22.80%	20.60%	21.80%	22.00%	21.40%	1	27.60%	27.70%
8	66.06%	22.30%	23.50%	21.80%	21.80%	22.40%	84.02%	1	67.40%
9	66.34%	22.30%	22.90%	20.40%	20.80%	23.60%	84.08%	95.98%	1

1, *C. gastrosuis* sp. nov.; 2, *C. mucosalis*; 3, *C. majalis*; 4, *C. suis*; 5, *C. pinnipediorum* subsp. *caledonicus*; 6, *C. pinnipediorum* subsp. *pinnipediorum*; 7, *C. coli*; 8, *C. jejuni* subsp. *doylei*; 9, *C. jejuni* subsp. *jejuni*; The sequence used here is the same as in the phylogenomic analysis.

**Table 3:** MICs of novel strain PS10^T^ to antimicrobials.

Antimicrobial Class	Antimicrobial Agent	MIC (μg mL^−1^)
Macrolides	Erythromycin	>256
	Azithromycin	>256
Quinolones	Nalidixic acid	>256
	Ciprofloxacin	3
Aminoglycosides	Gentamicin	3
	Streptomycin	8
Chloramphenicol	Chloramphenicol	12
	Florfenicol	8
Tetracyclines	Tetracycline	32
Ketolides	Telithromycin	>256
Lincosamides	Clindamycin	>256

**Table 4:** Description of *Campylobacter gastrosuis* sp. nov.

Genus name	*Campylobacter*
Species name	*Campylobacter gastrosuis*
Specific epithet	*gastrosuis*
Species status	sp. nov.
Species etymology	(gas.tro.su’is., Gr. n. gaster gastros, stomach; L. n. sus suis, a pig; L. gen. n. gastrosuis, from a pig’s stomach)
Description of the new taxon and diagnostic traits	The cell is Gram-negative, motile, and spiral-shaped after 48 h of growth on Karmali or Columbia agar with 5% defibrinated sheep blood in a microaerophilic atmosphere at 37 °C. The colonies are wet, flat, grey, circular, and smooth but may vary in size and morphology after a long incubation. No hemolysis on blood agar was observed. Cells are negative activities for catalase, oxidase, and urease, and could not produce H2S. No hydrolysis of hippurate. Nitrate can be reduced and indoxyl acetate can be hydrolyzed. The isolate was resistant to different types of antibiotics, namely erythro-mycin, azithromycin, nalidixic acid, tetracycline, telithromycin, and clindamycin, and carries multiple resistance relative genes and has an MDRGI.
Country of origin	China
Region of origin	Beijing
Source of isolation	the gastric mucous of pigs
Sampling date (dd/mm/yyyy)	14 March 2022
Latitude (xx°xx′xx″ N/S)	116°18′40″ N
Longitude (xx°xx′xx″ E/W)	40°11′27″ E
Altitude (meters above sea level)	About 30 m
16S rRNA gene accession nr.	OP278864
Genome accession number	JANURM000000000
Genome status	Draft
Genome size	2240 kbp
GC mol%	37.72
Designation of the Type Strain	PS10^T^
Strain Collection Numbers	GDMCC 1.3686^T^; JCM 35849^T^

## Data Availability

The GenBank/EMBL/DDBJ accession numbers of the National Center for Biotechnology Information (NCBI, https://www.ncbi.nlm.nih.gov/) (accessed on 20 June 2023) for the nearly full-length 16S rRNA gene and the draft genome sequences of this isolate PS10^T^ are OP278864 and JANURM000000000, respectively.

## Supplementary Materials

The following supporting information can be downloaded at: https://www.mdpi.com/article/10.3390/microorganisms11092278/s1, Figure S1: Photomicrograph of Gram-stained exponentially growing *Campylobacter gastrosuis* sp. Nov. PS10^T^ cells. A light microscope with 100× magnification was used; Figure S2: Maximum-likelihood phylogenetic tree based on nearly complete 16S rRNA gene showing the relationships between PS10^T^ and the type strains of the genus *Campylobacter*. Bootstrap values (>70%) based on 1000 replicates are shown at branch nodes, with *Arcobacter butzleri* ATCC 49616^T^ as an outgroup. Novel strain is highlighted in bold; Figure S3: Maximum-parsimony phylogenetic tree based on nearly complete 16S rRNA gene showing the relationships between PS10^T^ and the type strains of the genus *Campylobacter*. Bootstrap values (>70%) based on 1000 replicates are shown at branch nodes, with *Arcobacter butzleri* ATCC 49616^T^ as an outgroup. Novel strain is highlighted in bold. Table S1: ANI (lower diagonal) and dDDH (upper diagonal) among the novel Campylobacter strains and other Campylobacter species.

## Abbreviations

ANI, average nucleotide identity; dDDH, digital DNA-DNA hybridization; ML, maximum-likelihood; MP, maximum parsimony; NJ, neighbor-joining; GBS, Guillain–Barré Syndrome; MDRGI, multi-drug resistance genomic island; COGs, Clusters of Orthologous Groups; WGS, whole genome sequencing; HGT, horizontal gene transfer; MGEs, mobile genetic elements.
