# Supplementary figures and images for: Phenotypic and Genomic Characteristics of Campylobacter gastrosuis sp. nov. Isolated from the Stomachs of Pigs in Beijing
Source: Microorganisms. 2023 Sep 10;11(9):2278. doi: 10.3390/microorganisms11092278 (PMC10534318; doi:10.3390/microorganisms11092278)

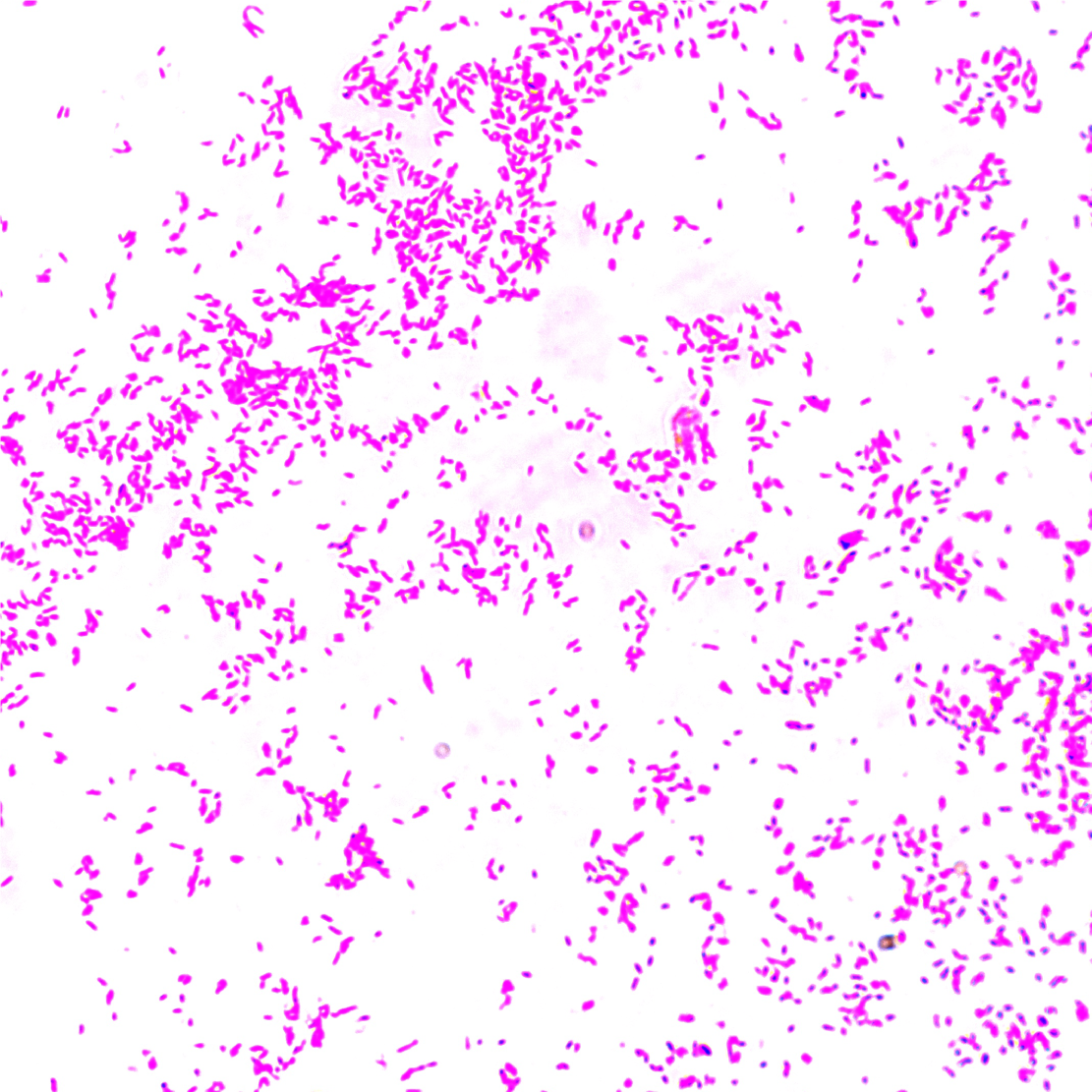

Supplement: Supplementary file 1 [file microorganisms-11-02278-s001.zip › Figure S1.tif]

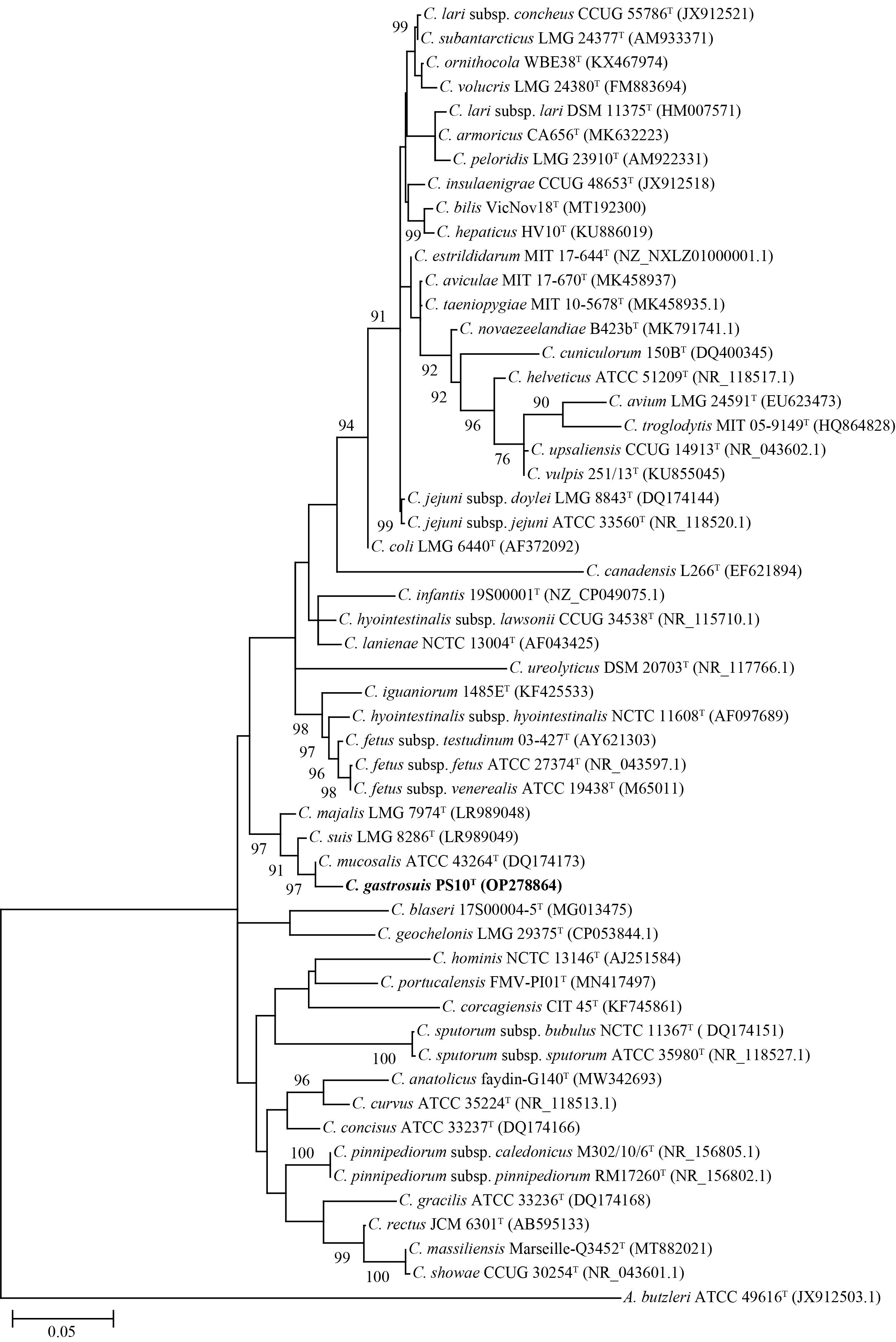

Supplement: Supplementary file 1 [file microorganisms-11-02278-s001.zip › Figure S2.tif]

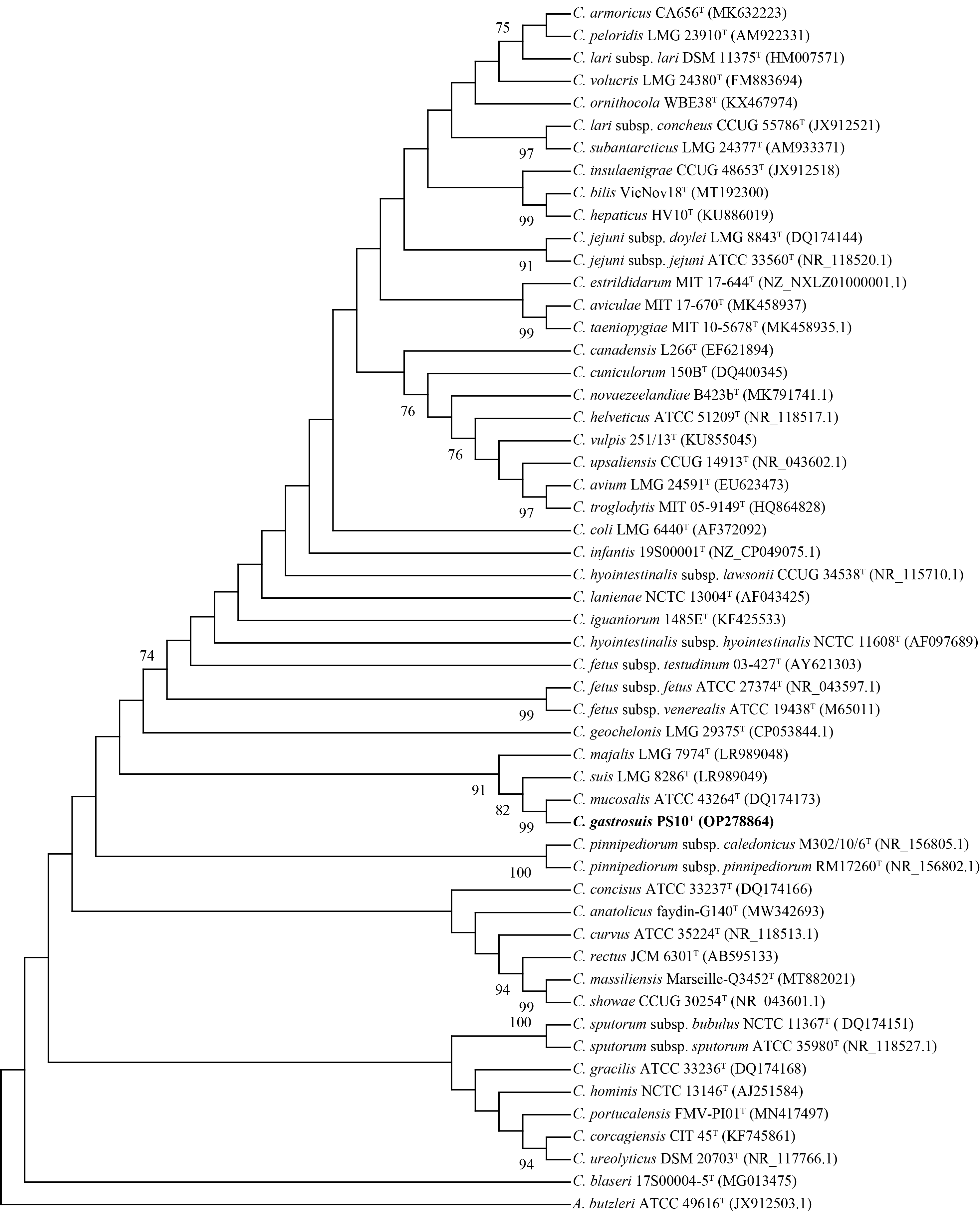

Supplement: Supplementary file 1 [file microorganisms-11-02278-s001.zip › Figure S3.tif]
